# Draft Genome Sequence of an Onion Basal Rot Isolate of *Fusarium proliferatum*

**DOI:** 10.1128/MRA.01385-18

**Published:** 2019-01-03

**Authors:** Andrew D. Armitage, Andrew Taylor, Michelle T. Hulin, Alison C. Jackson, Richard J. Harrison, John P. Clarkson

**Affiliations:** a NIAB EMR, Kent, United Kingdom; b Warwick Crop Centre, School of Life Sciences, University of Warwick, Wellesbourne, Warwick, United Kingdom; Vanderbilt University

## Abstract

Fusarium proliferatum is a component of the onion basal rot disease complex. We present an annotated F. proliferatum draft genome sequence, totaling 45.8 Mb in size, assembled into 597 contigs, with a predicted 15,418 genes. The genome contains 58 secondary metabolite clusters and homologs of the Fusarium oxysporum effector SIX2.

## ANNOUNCEMENT

Fusarium proliferatum is a generalist pathogen of a range of crops as diverse as maize, pineapple, and asparagus (1). It is also a component of the onion basal rot complex alongside the more common pathogen Fusarium oxysporum f. sp. cepae and may cause discoloration of onion bulb scales (2–5). Genomic resources for F. oxysporum from onion, alongside those for F. proliferatum strains isolated from other crops, have recently become available (6–8). Expansion of these resources with a genome sequence of F. proliferatum from onion provides a basis for a study of host adaptation within F. proliferatum and for different strategies of onion infection within the *Fusarium* genus.

F. proliferatum isolate A8 was isolated from an onion bulb with basal rot symptoms obtained from a commercial grower in Bedfordshire, United Kingdom, in 2009. DNA extraction was performed on freeze-dried mycelium using a Macherey-Nagel NucleoSpin plant II kit (catalog no. 11912262; Fisher). Paired-end (PE) genomic libraries were then prepared using an Illumina TruSeq LT kit (catalog no. FC-121-2001). Libraries were sequenced using a 2 × 250-bp PE kit (catalog no. MS-102-2003) on an Illumina MiSeq version 2 instrument, generating 2,986,704 paired reads.

Reads were trimmed and adapters removed using fastq-mcf version 1.04.676 (9) before a 45.8-Mb genome assembly was generated using SPAdes version 3.5.0 (10) in 581 contigs (Table 1). Repeat masking was performed using RepeatMasker version 4.0.3 (http://www.repeatmasker.org) and TransposonPSI (http://transposonpsi.sourceforge.net, 2013-03-05 release). This masked 1.21 Mb of the genome. Genome completeness was assessed through the presence of conserved single-copy fungal genes using BUSCO version 3 (11). We used the Sordariomyceta *odb9* data set, identifying 3,694 of 3,725 (99%) of these genes as present in the assembly. Published RNA sequencing (RNA-seq) data (7) were aligned to the genome using Bowtie 2 version 2.2.4 and TopHat version 2.1.0 (12, 13), with mate-inner-dist set to −20 bp and mate-std-dev set to 70 bp. These alignments were used in the prediction of 15,418 genes encoding 15,448 proteins; 15,421 of these proteins were predicted using BRAKER1 version 2.0 (14), supplemented by an additional 37 proteins predicted by using CodingQuarry version 2.0 (15), located in intergenic regions of BRAKER1 gene models. BRAKER1 was run using the fungal option, and CodingQuarry was run in pathogen mode. Functional annotation was performed using InterProScan version 5.18-57.0 (16) and through BLASTp (E value < 1 × 10^−100^) searches against the July 2016 release of the Swiss-Prot database (17).

**TABLE 1:** *F. proliferatum* isolate A8 genome statistics

Statistic	Value
Assembly statistics	
Assembly size (bp)	45,689,467
No. of contigs	581
Largest contig (bp)	1,926,525
GC content (%)	48.65
*N*_50_ (bp)	535,935
% repeat masked	2.65
Gene models	
Total no. of genes	15,418
Total no. of proteins	15,458
No. encoding secreted proteins	1,254
No. of genes encoding effector candidates:	
Secreted and effector-like structure	258
Secreted CAZYmes^*a*^	341
Secondary metabolites	
No. of gene clusters	58
mimp sequences	
No. of mimp sequences in genome	6
No. of genes in 2 kb of mimp sequences	11
No. of genes in 2 kb of mimp sequences encoding secreted proteins	1

a Carbohydrate-active enzymes.

tBLASTx searches (E value < 1 × 10^−10^) for pathogenicity factors associated with *Fusarium* spp. identified two homologs of the SIX2 gene from F. oxysporum f. sp. lycopersici on contigs 12 and 246 (18, 19). Additional pathogenicity factors were identified following the same approaches used for our recent annotation of F. oxysporum f. sp. cepae genomes (7) (Table 1). Secondary metabolite gene clusters were predicted using antiSMASH (20), SignalP version 4.1 and TMHMM version 2 were used to predict genes encoding secreted proteins (21, 22), carbohydrate-active enzymes were predicted using dbCAN and the CAZY database classifications (23, 24), and proteins with an effector-like structure were predicted using EffectorP version 1.0 (25). Furthermore, miniature impala (mimp) sequences are located in the vicinity of F. oxysporum f. sp. genes that are important in pathogenicity (26) and as such were identified in the genome (Table 1) using previously described RegEx searches (7).

### Data availability.

This whole-genome shotgun project has been deposited at DDBJ/ENA/GenBank under the accession number MRDB00000000 (BioProject number PRJNA338256). The version described in this paper is version MRDB01000000. Reads are available from GenBank under accession number SRR4408423.
